# Production of Gynogenic Plants of Red Beet (*Beta vulgaris* L.) in Unpollinated Ovule Culture In Vitro

**DOI:** 10.3390/plants10122703

**Published:** 2021-12-08

**Authors:** Tatyina Zayachkovskaya, Elena Domblides, Vladimir Zayachkovsky, Lyudmila Kan, Arthur Domblides, Alexey Soldatenko

**Affiliations:** Federal State Budgetary Scientific Institution Federal Scientific Vegetable Center (FSBSI FSVC), VNIISSOK, 143072 Moscow Region, Russia; vladimir89854217114@mail.ru (V.Z.); loyus@mail.ru (L.K.); arthurdom@inbox.ru (A.D.); alex-soldat@mail.ru (A.S.)

**Keywords:** red beet, unpollinated ovule culture in vitro, haploid, induction medium for *Beta vulgaris* (IMB), DH plants, homozygous lines, induction of embryogenesis, regeneration, ploidy level

## Abstract

The unique and balanced components of the biochemical composition, together with high antioxidant activity, make the red beet necessary a dietary vegetable crop, much contributing to healthy food ration. The application of the technology for producing gynogenic plants in vitro increases the genetic diversity and significantly reduces the period of time required to obtain the appropriate homozygous lines used to create the F1 hybrids that are demanded in the market. For induction of gynogenesis, we used IMB medium developed by us with the addition of 55 g/L sucrose, 3 g/L phytogel, 200 mg/L ampicillin, and 0.4 mg/L thidiazuron (TDZ) and cultured at 28 °C in the dark for 4–6 weeks. Shoot regeneration from embryoids and callus was performed on MS medium with 20 g/L sucrose, 3 g/L phytogel, 1 mg/L 6-benzylaminopurine (BAP), and 0.1 mg/L gibberellic acid (GA_3_). Immersion of the obtained microshoots with 5–7 well-developed leaves for 10–15 s into concentrated sterile indole-3-butyric acid (IBA) solution (50 mg/L) followed by their cultivation on solid medium ½ _IMB_ with 2% sucrose and 3 g/L phytogel was the most efficient method for root formation. The addition of silver nitrate (22 mg/L) to the nutrient medium provoked an increase in the number of induced ovules up to nine per Petri dish (up to 25% of induced ovules). Gynogenic development was produced in six out of 11 genotypes studied, and the plants that were then acclimatized to ex vitro conditions were obtained in three genotypes (Nezhnost’, Dobrynya, b/a 128). The evaluation of ploidy of gynogenic plants that was carried out by flow cytometry and direct counting of chromosomes stained with propion-lacmoide revealed that all obtained gynogenic plants were haploids (2n = x = 9).

## 1. Introduction

Red beet (*Beta vulgaris* L. ssp. *europaea* Krass. var. *atrorubra* Krass.) is a biennial, cross-pollinated plant of the Amaranthaceae family (Chenopodiaceae Vent.); it is one of the main vegetable crops in Russia and is widely distributed in Central, Western, and Southern Europe and Asia.

Red beetroot is especially popular and widespread due to its early maturity, high yield, and valuable biochemical composition, optimal contents of carbohydrates, mineral salts, organic acids, and vitamins. Moreover, it contains vitamins C, B1, B2, B6, PP, P, calcium, magnesium, iron, malic, citric, oxalic, lactic, organic acids, along with biotin, folic, and pantothenic acids. Importantly, phosphorus and potassium are contained in red beets in the most favorable ratio for humans. The special value of beets is that it contains more salts than acids. Beetroot juice contains large amounts of betaine (trimethylglycine or glycinebetaine), which is not found in other vegetable plants. Betaine promotes better digestion of food and is involved in the formation of choline, a substance that improves liver cells, strengthens capillaries, and reduces the accumulation of cholesterol in the blood. Beets are also a major source of betalains, water-soluble nitrogen pigments with a heterocyclic ring that can be divided into two classes based on their chemical structure: (1) betaxanthins (responsible for orange-yellow coloration) and (2) betacyanins (responsible for red-purple coloration) [1]. The most abundant betacyanin contained in red beetroot, in large quantities, is betanin (betanidin 5-O-β-d-glucoside), which is medically proven to be inhibitory to malignant tumor growth [2,3]. The unique and balanced composition of red beet, along with its high antioxidant activity, afford red beet its health-promoting status [4]. Due to its anti-inflammatory, cognitive, anti-cancer, and anti-hepatitis properties, betalains contained in beetroot can be useful as pharmaceutical agents and dietary supplements [5]. In addition, betanin is the only component approved for use as a natural colorant in food products, cosmetics, and pharmaceuticals, under code E162 by the European Union [6] and the U.S. Food and Drug Administration (FDA) [7,8].

Another advantage of red beetroot is its long-term storage, which preserves its beneficial properties and nutritional and dietary value, which contribute to the year-round fresh-form consumption of the product. Beetroot consumption begins in early spring in the form of young leaves and petioles, grown in protected conditions from preserved root crops from autumn; in the summer months, young plants are used as food, and in the autumn–winter period, they are used as preserved root crops. Root crops are widely used fresh, boiled, canned, dried, and sun-dried [9,10,11].

According to the Agribusiness Expert-Analytical Center, red beet in the Russian vegetable production industry, in 2020, accounted for 32.7 thousand hectares of sown area and 801 thousand tons of marketable products [12]. However, the leader in this family, in terms of prevalence, sown areas, and marketable products, is sugar beet (*Beta vulgaris* L. ssp. *europea* Krass. var. *saccharifera* Alef.), the most important technical crop that is grown for sugar production and other processing by-products, including molasses used in the food, chemical, and perfume industries and pulp, which is used as feed for farm animals and pectin and glue production. Indeed, because of this crop, new technologies have been extensively developed [13].

One of the most promising technologies currently being intensively developed in many countries around the world and which allows significant acceleration of the breeding process is doubled haploids (DH) technology. The advantage of using doubled haploids in practical breeding is determined by their complete homozygosity, absence of dominance effect, detection of rare recessive alleles, and the expansion of the range of form-forming processes due to recombinant variability, all contributing to the creation of unique forms and increasing the efficiency of practical breeding. In addition, it takes 1–2 years to create a line of doubled haploids, while it takes 8–10 years to obtain a homogeneous line using classical breeding methods. The process of creating doubled haploids consists of two steps: (1) obtaining a haploid embryoid/callus and (2) the subsequent chromosome doubling [14,15].

The first haploid plant in *B. vulgaris* species was detected and described in the analysis of seed progeny of sugar beet obtained in experiments using colchicine treatment of shoots [16]. Later, haploids were found in the progeny of plants treated with polyploidizing agents: among twin seeds isolated from diploid or anisoploid varieties, among the progeny of diploid cytoplasmic plants with male sterility, among spontaneous twins and plantlets produced by irradiated pollen, and by distant crosses (Zimmermann (1953) [17], Fischer (1956) [18], Butterfass [19], Kruse (1961) [20], Dobretsova et al., (1965) [21] and Hammond (1966) [22]; Bosemark (1971) [23]). However, for a long time, haploid sugar beet was not used in breeding since traditional methods allowed selecting only a limited number of haploid sugar beet plants [24]; moreover, there were no protocols for multiplication and maintenance of haploid plants in in vitro culture, for transfer to the diploid level.

From the development of the induced haploid production technique in 1964, using anther culture in tobacco [25], to the initial production of haploid callus from the female gametophyte of *Ginkgo biloba* L. [26], and the more successful production of the first haploid plants in an unpollinated ovule culture of *Hordeum vulgare* [27], the production of haploid plants (based on in vitro cultivation techniques of male and female gametophytes) has been introduced into breeding practice for other crops.

Initial attempts at induced production of doubled haploids of sugar beet in in vitro culture were made using male gametophytes. However, cultivation of anthers was not successful, and it was not possible to obtain androgenic plants; only the formation of multicellular structures, callus, and roots was induced from microspores [28,29]. The first successful cultivation of female gametophytes to produce haploid and doubled haploid sugar beet plants using an in vitro unpollinated ovule culture was reported by Hosemans and Bossoutrot (1983) [30], D’Halluin and Kelmer (1986) [31], and Van Geyt et al. (1987) [32]. Since then, and until now, various in vitro tissue culture techniques, genetic transformation, molecular biology techniques, in vitro selective systems (contributed to obtaining sugar beet plants with high tolerance to abiotic stresses) have been used to improve the characteristics of this crop, propagation, and conservation of valuable forms in practical breeding [33,34,35,36].

Despite a large number of experimental approaches, including those based on the creation of haploid sugar beet lines using mutant *cenh3* haploid inducer lines [37], doubled haploids of sugar beet are obtained mainly using in vitro unpollinated ovule cultures. The obtained DH lines are used in breeding and seed production processes to create sugar beet hybrids in many European firms [33]. The development of this technique is being carried out in Germany [38], Sweden [39], Russia [33], Belarus [40], Serbia [41], Denmark [42], Turkey [43,44,45], Poland [46], and Iran [47]. However, the efficiency of gynogenesis induction in sugar beet remains negligible in most scientific studies (ranging from 1% to 15%), and the efficiency of regenerant yield is 40% [39]. Importantly, there are isolated data on the high efficiency of gynogenesis (37.8%), but only for a limited selection of genotypes [44].

At present, the economic importance of red beet requires the introduction of modern biotechnological methods into the breeding process with the possibility of obtaining doubled haploid plants for accelerated synthesis of high-yield hybrids. There is limited, sporadic literature data regarding the application of the gynogenesis and androgenesis method in red beet [48,49]. In addition, these publications provide only data on the induction of the gynogenesis process in in vitro unpollinated ovule culture and androgenesis in microspore culture; however, no information exists regarding obtained DH plants of red beet. In 2021, a protocol for obtaining doubled haploids of red beet by in vitro gynogenesis was generated [50]; however, its efficiency remains to be tested. In this regard, the creation of a highly efficient, reproducible technology to produce doubled haploids of red beet is an urgent task for world breeding programs.

## 2. Results

### 2.1. Induction of Gynogenesis in an Unpollinated Ovule Culture of Red Beet In Vitro

**Figure 1:**
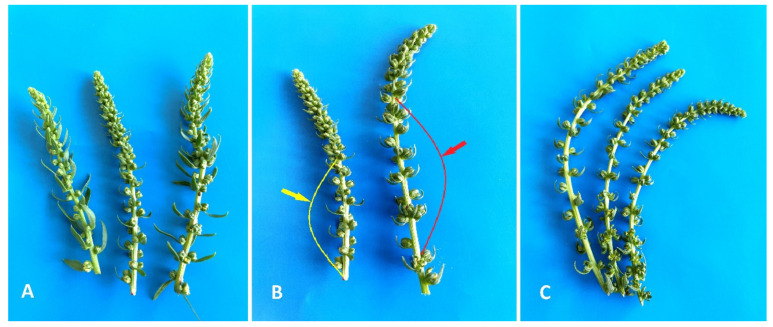
Inflorescences of red beet: (**A**) cv. Nezhnost’; (**B**) marked sections of inflorescences selected for in vitro cultivation (yellow arrow—cv. Nezhnost’, red arrow—cv. Dobrynya); (**C**) cv. Dobrynya.

The inflorescence of red beet is a loose spike with buds that can be arranged singly or in groups of 2–3 (depending on the genotype). In the apical part of the spikelet inflorescence, flowers are densely arranged from one to another, and as the inflorescence develops, the distance between the buds increases, their size increases, and in the later stages of development, the buds locate to the basal part of the inflorescence. From the literature, it is known that in unpollinated sugar beet ovules cultures, the optimal stage for in vitro introduction is comma-shaped ovules containing an almost mature or fully mature embryo sac. In our study, only comma-shaped red beet ovules with a length of 1–3 mm, corresponding to the selection of 8–10 buds on the spike-like peduncle section above the opened flower, 2–6 cm long (depending on the genotype) to the section where the distance between the buds or a group of buds is less than the length of the buds, were used for introduction into a culture. The length of the selected inflorescence section for selecting buds from donor plants of different genotypes varied. For example, the larger buds of the red beet cv. Dobrynya were located farther apart from each other on the inflorescence axis than in the cv. Nezhnost’ variety. This, accordingly, influenced the size of the selected inflorescence area when selecting buds before introduction into in vitro culture (Figure 1).

Over the course of the study, before introducing explants to in vitro culture, we selected a stepwise surface sterilization regime using a 96% alcohol/5% sodium hypochlorite solution, which, combined with the addition of ampicillin at a concentration of 200 mg/L to the culture medium, ensure 100% yield of uninfected viable explants.

**Figure 2:**
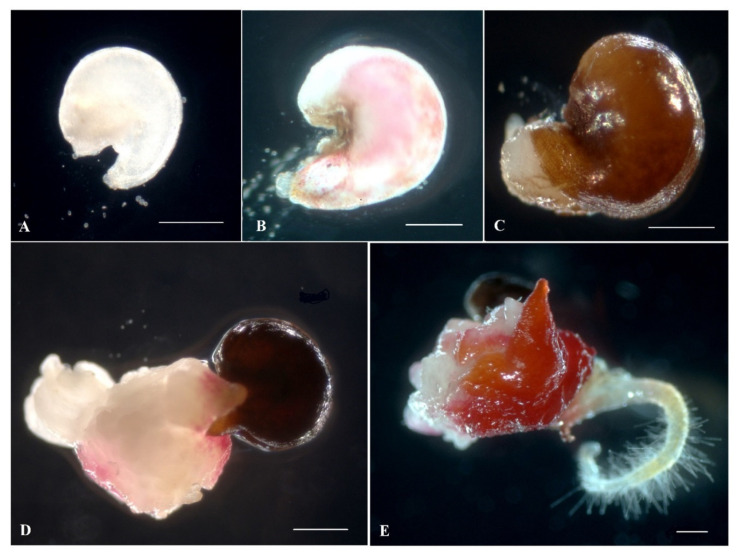
Embryoid/callus induction on IMB medium with 0.4 mg/L TDZ in a culture of unpollinated red beet ovules: (**A**) isolated ovule at a suitable stage of development immediately after isolation; (**B**) ovules at the third day of cultivation; (**C**) ovules at 35 days of cultivation; (**D**) rupture in the micropillar part of the ovules with callus formation; (**E**) embryoid structure developing from the ovules after six weeks of cultivation. Bars = 1000 µm.

**Figure 3:**
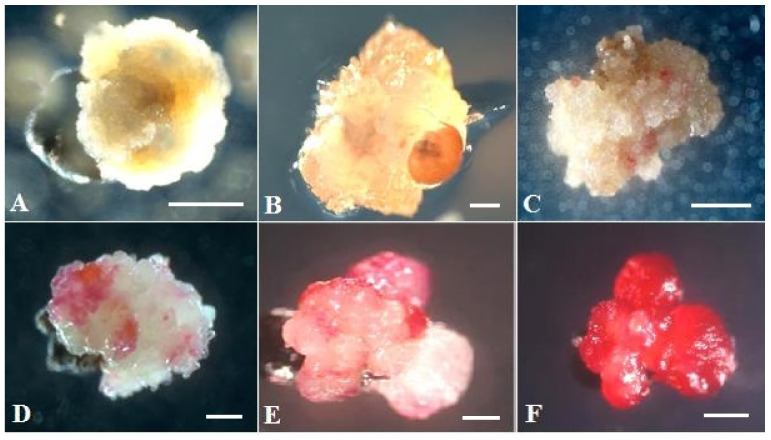
Formation of different types of morphogenic callus from induced red beet ovules of different genotypes on IMB medium with 0.4 mg/L TDZ: (**A**,**E**,**F**) b.a. 128; (**B**,**C**) b.a. 135; (**D**) cv. Dobrynya. Bars = 1000 µm.

As early as the third day after introducing the ovules into the in vitro culture, a slight increase in size and color change from white to pink were observed (Figure 2A,B). After three weeks of cultivation, the ovules turned fulvous in color, turning brown after another week. From the induced ovules after four to six weeks of cultivation, primary callus or embryoid formation was observed (Figure 2D,E). Initially, under a stereomicroscope, a rupture of the shell of the induced ovule in the micropillar end could be detected, from which dense white tissue/white contents protruded (Figure 2C). The resulting callus (Figure 2D) or embryoids then increased in size quite rapidly (within a week) and became clearly visible to the naked eye (Figure 2D,E). Gynogenic development through embryoids and through callus was observed in three genotypes, (cv. Nezhnostʹ, cv. Dobrynya, and b.a. B-131), in the other three (b.a. P-155, b.a. 135, b.a. 128), development was observed only through callus formation. The callus tissue color in all genotypes varied from white, yellowish, white-pink, partially pigmented with anthocyanins, to intensely red with full anthocyanin pigmentation (Figure 3). Regardless of the coloring and consistency of the callus, it was possible to initiate development along the organogenesis pathway at the regeneration stage from all callus types.

In the experiment, it was possible to induce callus formation in 6 of the 11 red beet genotypes included in the study. Depending on the genotype, the average number of gynogenesis-inducing ovules ranged from 1.2 ± 0.37 to 7.4 ± 0.23 per Petri dish (Table 1). In the three genotypes, a maximum of six to nine induced ovules per Petri dish was obtained. The maximum responsiveness to gynogenesis induction was observed in the Nezhnost’ red beet cultivar. In the five genotypes studied, no inducible gynogenesis activity was detected. A one-factor ANOVA analysis of variance confirmed a significant effect of genotype on gynogenesis induction in an in vitro unpollinated ovule culture.

### 2.2. Effect of Silver Nitrate on Induction Capacity of Red Beet in an Unpollinated Ovule Culture In Vitro

**Figure 4:**
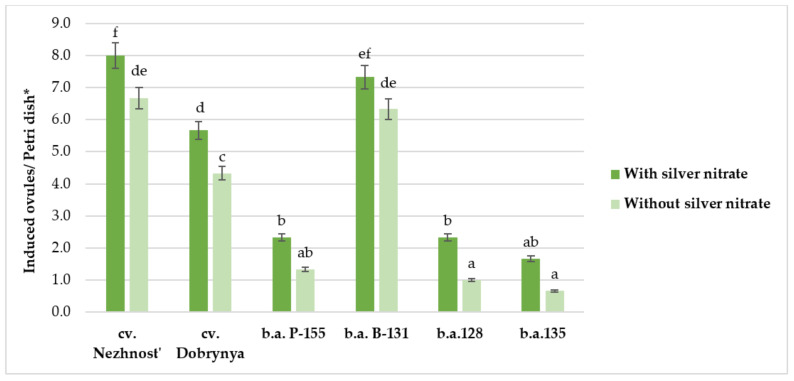
Effect of silver nitrate (22 mg/L) on induction capacity of red beet ovules of different genotypes on IMB medium with 0.4 mg/L TDZ.Note: * For each genotype, the average values with five repeats in each ± SE (standard error) are presented. Two-factor analysis of variance (ANOVA) was used, and the average values were compared using Student’s *t*-test with 95% probability. Significant difference: Factor A (genotype): Fobserved 87.08 > Ftheor. 2.62; Factor B (silver nitrate): Fobserved 23.21 > Ftheor. 4.25. Values marked with similar letters had no significant difference at *p* ≤ 0.05. LSD = 1.2.

In experiments with the addition of silver nitrate (22 mg/L) to the induction medium, an increase in the number of induced ovules was observed for all genotypes. Thus, cv. Nezhnost’, the most responsive at the induction stage, had a maximum of nine induced ovules per Petri dish with a diameter of 10 cm, containing 35 ovules, an increase of 19% compared to the variant without silver nitrate addition. The genotypes that were responsive at the induction stage (cv. Nezhnost’, cv. Dobrynya and b.a B-131) had an excess of 19%, 33%, and 16%, respectively. In the less responsive varieties at the induction stage (b.a. 128 and b.a. 135), the addition of silver nitrate contributed to a 2.3- and 2.4-fold increase in induced ovules, respectively (Figure 4). Moreover, it was observed that in the presence of silver nitrate, the ovules did not change their color to dark brown for a longer duration and remained white or pale pink. ANOVA confirmed a significant effect of genotype and silver nitrate on the induction of gynogenesis in in vitro culture. The effects from genotype and silver nitrate factors were 90% and 4%, respectively. At the regeneration stage, there was no difference between the developing embryoids/callus induced on media with and without silver.

### 2.3. Plant Regeneration

**Figure 5:**
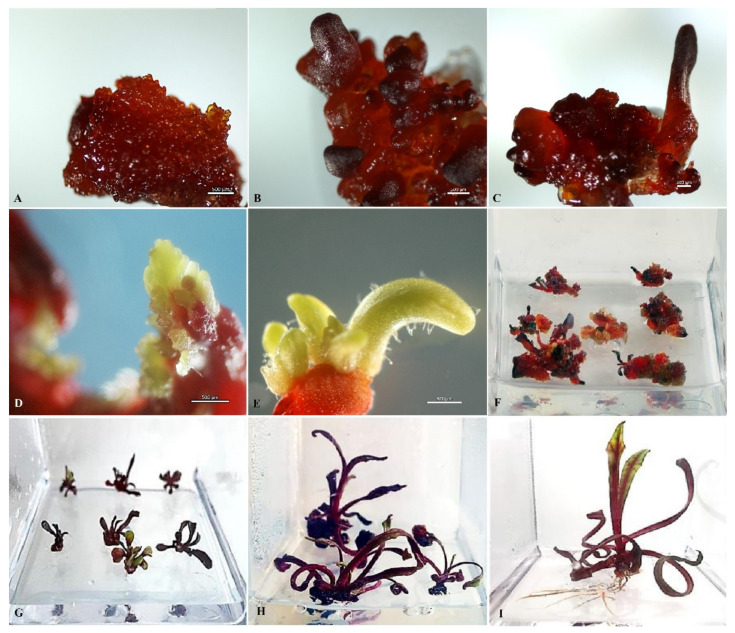
Plant regeneration in red beet unpollinated ovule culture: (**A**) morphogenic callus developing from induced ovule after four to six weeks of cultivation; (**B**,**C**) formation of green meristematic nodes in callus initiating shoot regeneration; (**D**–**F**) shoot regeneration; (**G**,**H**) development of red beet regenerant plants; (**I**) formation of root system in regenerant plants under in vitro conditions.

Direct germination and formation of microrosettes occurred when the embryoid was placed on regenerating nutrient medium with 1 mg/L BAP and 0.1 mg/L GA_3_. However, the root system was quite weakened, and there was an overgrowth of the cotyledon and hypocotyl sites with the formation of additional microrosettes. After a series of successive separations of the microrosettes and transplanting to fresh nutrient hormone-free medium, the formation of large rosettes with 5–7 well-developed leaves (ready for transplanting to nutrient medium for rooting) was achieved (Figure 5E). The embryoids were present in the three examined genotypes. The highest number of ovules inducing development through the embryoid was observed in the cv. Nezhnost’ variety and accounted for 24% of the total number of induced ovules (Table 2).

In the case of gynogenic development through callogenesis observed in all studied genotypes, the resulting callus was transferred to light on regenerating nutrient medium, sometimes without separating it from the ovules (Figure 5A). Four of the six genotypes were able to initiate regeneration and produce microrosettes/microshoots (Table 2).

**Figure 6:**
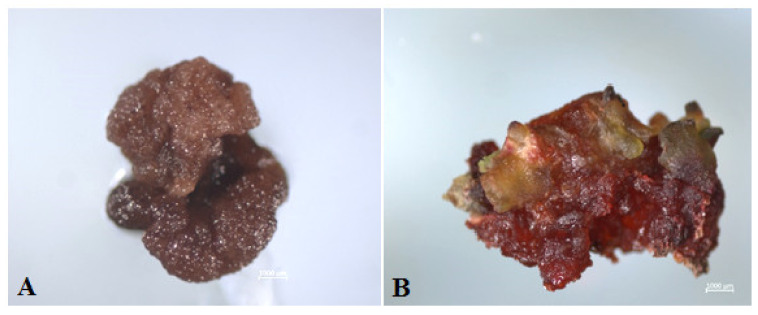
Inhibition of callus development: (**A**) with early transfer to light to regenerating nutrient medium; (**B**) with no change of nutrient regenerating medium for more than three weeks.

A critical factor that determines the success of regeneration was the size of the callus fragments cultured in the dark and transferred to light conditions on the regeneration nutrient medium; when the callus is smaller than 5 mm, it usually degrades. (Figure 6A). Successive subcultures of primary callus on regeneration nutrient medium with 1 mg/L BAP and 0.1 mg/L GA_3_ should be performed in order to prevent senescence, loss of division, and further growth and shoot formation from meristematic green nodes and callus cell death in responsive genotypes (Figure 6B). Once microshoots were formed on the callus surface, they had to be separated and replanted on fresh nutrient medium every two to three weeks until viable regenerants were formed (Figure 5D).

### 2.4. Rooting of Regenerant Plants, Derived from Unpollinated Ovule Culture In Vitro

Since the majority of well-developed, non-vitrified microshoots obtained in the regeneration process were from the cv. Nezhnost’ variety, they were used in experiments for the selection of an optimal nutrient medium for rooting. Microrosettes with well-developed 5–7 leaves were transferred to three variants of nutrient medium (Table 3).

After four weeks of culture, the use of MS media without plant growth regulators resulted in rooting of only 5% of the microshoots. Further cultivation on this nutrient medium with transfer to fresh nutrient medium every two weeks did not increase the number of rooted shoots even after 8 weeks of cultivation. Using ½ IMB nutrient medium supplemented with 2 mg/L IBA increased the rate of rooted shoots to 25% by the fourth week of cultivation.

**Figure 7:**
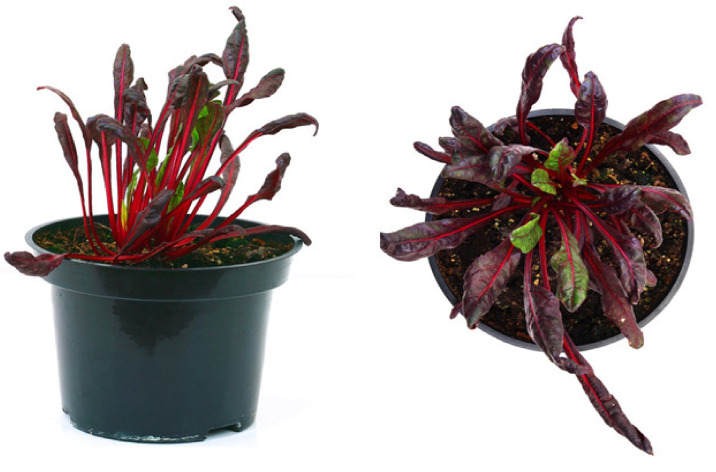
Ex vitro acclimatized haploid regenerated plant of red beet cv. Nezhnost’ obtained in unpollinated ovule culture in vitro.

Only immersion of microrosettes for 10–15 s in IBA solution (50 mg/L) before transferring to solid ½ IMB medium without plant growth regulators, after two weeks, stimulated root development in 80% of explants. To form a well-developed root system, a second immersion in concentrated IBA solution and transplantation to fresh nutrient medium was performed after two weeks (Table 3). The rooting method with immersion in sterile IBA solution (50 mg/L) followed by cultivation on solid ½ IMB medium without plant growth regulators was further used to root the obtained microshoots in genotypes Dobrynya and b.a. 128. Plants with a well-developed root system (Figure 5I) could be planted in pots with a mixture of peat and perlite with the obligatory use of perforated plastic cups. The adaptation period of the regenerant plants to ex vitro conditions was significantly long-amounting to two months (Figure 7). Out of 44 plants of three genotypes (Nezhnost’, Dobrynya, and b.a. 128) planted, only 15 plants survived. Therefore, the losses at the adaptation stage to ex vitro conditions averaged 65.9%.

### 2.5. Determination of Ploidy of Regenerant Plants, Obtained in Unpollinated Ovule Culture

**Figure 8:**
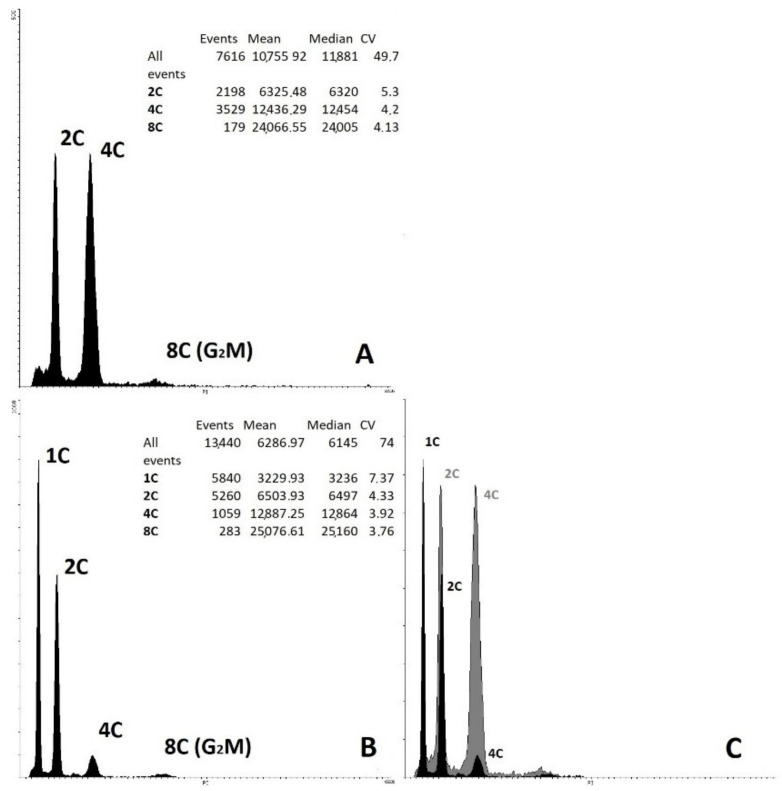
Histogram of red beet samples, obtained in an unpollinated ovule culture in vitro. External standardization (study without changing cytometer settings). (**A**) Control diploid sample cv. Nezhnost’; (**B**) Haploid regenerated plant; (**C**) Combined histogram of the haploid sample (black) and diploid control (gray).

**Figure 9:**
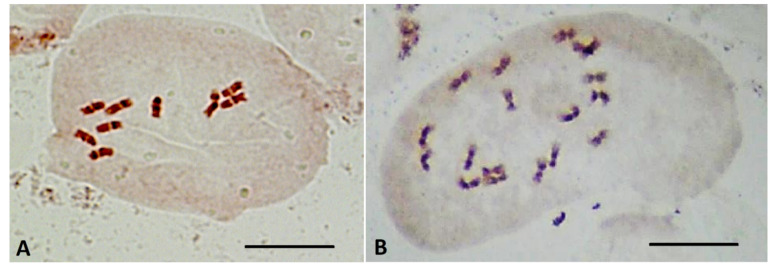
Chromosomes in mitotic metaphases of shoot apical meristems of red beet: (**A**) haploid regenerant plant (2n = x = 9), obtained in an unpollinated ovule culture in vitro; (**B**) diploid plant (2n = 2x = 18). Bars = 10 µm.

The ploidy of the regenerated plants was confirmed using flow cytometry of cell nuclei (Figure 8) and direct chromosome counting in apical meristems (Figure 9). The haploid ploidy level was detected in 100% of 15 plant regenerants that successfully passed the acclimatization stage to ex vitro conditions, regardless of whether they were obtained through embryoids or callogenesis.

Ploidy-level cytophotometric evaluation of red beet regenerants obtained in in vitro unpollinated ovule culture revealed haploid forms with a single (2n = x = 9) set of chromosomes, indicating their origin from haploid cells of the embryo sac. All analyzed samples were characterized by endopolyploidy (appearance of three or more peaks) (Figure 8). Most haploid plants also showed a slight presence of diploid nuclei. Karyological analysis, using the propion-lacmoid method of chromosome staining in apical meristem cells of red beetroot, confirmed the haploid set of chromosomes in the obtained regenerant plants (Figure 9). Differences in the chromosome set were also evident in the obtained regenerants morphologically: haploid forms had narrow leaves, greater foliated leaf rosettes (Figure 7), and a poorly developed root system.

## 3. Discussion

The main factors affecting the induction and development of regenerant plants in unpollinated ovule culture were: (1) genotype and growth conditions of donor plants, (2) stage of ovule development, (3) pretreatment of buds, (4) composition of nutrient medium, including the ratio of certain growth regulators, and (5) in vitro cultivation conditions. The study and development of these factors have made it possible to create protocols for the production of doubled haploids using the gynogenesis method in many economically important agricultural plants, e.g., durum wheat [51], onion *Allium cepa* L. [52,53], cucumber *Cucumis sativus* [54], summer squash *Cucurbita pepo*, winter squash *C. maxima*, pumpkin *C. moschata* [55], and maize [56].

Currently, among crops belonging to the *Amarantheceae* family, the most numerous and successful studies have been those devoted to the study of gynogenesis in sugar beet. Doubled haploids of this economically important crop have been obtained over the years [31,38,47,57,58,59,60], and protocols for obtaining Dh plants through in vitro unpollinated ovule culture have been published [39,43,61]. Clearly, there is no universal methodology for obtaining doubled haploids, and although sugar beet and red beet belong to the same *Beta vulgaris* species, the developed protocols are not always successful due to high genotype specificity. Until recently, the only known study for red beet was that of Baranski (1996) [48], which examined some of the factors inducing gynogenesis [48]. The first protocol for obtaining doubled haploids of red beet in in vitro unpollinated ovule culture appeared in 2021 [50], whose efficacy for most genotypes has yet to be tested. Our study, initiated in 2018, focused on breeding valuable genotypes with significant demand in Russia and aimed at investigating gynogenesis and plant regeneration factors in red beet through in vitro unpollinated ovule culture. The choice of donor plants and the period and place of their growth during the year have a significant influence on the induction ability toward gynogenic development in in vitro unpollinated ovule culture. Evidence suggests that the most optimal time of donor plant growth for isolation of explants in sugar beet unpollinated ovule culture is from May to September [38,57]. It has been reported that there is no significant difference in seedling responsiveness between chamber-grown and greenhouse-grown plants with sufficient light in the growth chamber [38]. At the same time, experiments by Baranski (1996) [48] showed no difference between spring and summer growth of donor red beet plants; however, one experimental year showed a higher response of ovules collected from greenhouse-grown plants than from field-grown plants. In our studies (2019–2021), bud sampling was performed from donor plants of different greenhouse-grown red beet genotypes during the mass flowering period from April to July; however, we failed to identify any gynogenesis induction pattern from spring-summer seasons.

It is known that for successful induction of gynogenesis in all crops, the correct determination of the necessary female gametophyte development stage for introduction into in vitro culture plays an important role. According to the literature data, in sugar beet, the optimal stage for introduction into in vitro culture is ovules containing an almost mature or fully mature germinal sac. Hoseman and Bossoutrot (1983) [30], who first obtained 17 haploid plants from 7237 sugar beet ovules, reported in their studies that ovules containing a 7-core germ sac were most responsive in in vitro culture. This was later confirmed in several other works. Ferrant and Bouharmont (1994) [62] noted that sugar beet buds collected 1–3 days before flowering already contained fully mature gametophytes and were suitable for cultivation. In a study by Van Geyt et al. (1987) [32], only comma-shaped ovules were responsive to gynogenesis, while young spherical ovules degenerated after a few days of cultivation. Since it is not always possible to perform a cytological examination to determine directly the stage of the germ sac, the use of a parameter, such as the shape of the ovule, seems convenient; however, to isolate the necessary ovules, it is necessary to determine in which buds they will be contained. Therefore, it has been experimentally established that it is convenient to select a particular fragment of the inflorescence with reference to the first bud to opening. In our studies, with all 11 genotypes, we used only comma-shaped ovules, 1–3 mm in length, collected from a 2–6 cm long section of the spike-like peduncle immediately above the opening bud.

Many researchers noted changes in size and coloring of sugar beet ovule from white to brown after ovule isolation and its placement on induction nutrient medium [32]. The formation of gynogenic plants began from embryo-like structures developing from the micropillar part of the ovule [32,63]. In an in vitro unpollinated ovule culture of sugar beet, most haploid plants are formed by direct embryogenesis [64], whereas in our study in red beet, we observed both embryoid and callus formation, with the latter type predominating.

The composition and concentrations of nutrient medium components that require optimization, not only for each plant species but also for genotype, play an important role in modifying the induction activity in unfertilized ovule culture. So far, even for the most commonly researched sugar beet, there is no universally available nutrient media composition. In unfertilized ovules of sugar beet cultures, nutrient medium MS is most commonly used (Murashige and Skoog, 1962) [32,38,47,65], followed by N6 [57,62] and PG_0B_ [31] (Zhu and Wu, 1979) [66].

The choice of growth regulators (type and concentration) also has a significant influence on the induction ability of ovules. According to the literature, the cytokinin BAP is most commonly used for induction of gynogenesis in sugar beet. Lux et al. (1990) [38] reported the crucial importance of cytokinins for successful gynogenesis of sugar beet and increased embryoid yield when BAP is increased in the nutrient medium. In a study by Pazuki (2017) [44], the addition of BAP to the nutrient medium at a concentration of 2 mg/L doubled the induction activity to 10.75%. Gurel et al. (2000) [43] reported that the induction rate of sugar beet in media containing 1 mg/L or 2 mg/L BAP was 7.2% and 9.6%, respectively. Weich and Levall (2003) [39], in their protocol, recommended a combination of 1.33 µM BAP and 0.23 µM 2,4-D, 80 g/L sucrose for effective induction of sugar beet embryogenesis. Earlier sources reported that the rate of embryogenesis induction from sugar beet ovules cultured on hormone-free medium was 4–5%, compared to media supplemented with 1–2 mg/L BAP, in which induction was absent [31].

In a study by Baranski (1996) [48], the highest response of red beet ovules was found on nutrient medium N6 [67] with 0.5 mg/L IAA and 0.2 mg/L BAP [48]. A recently published protocol for producing gynogenic red beet plants in the induction stage used nutrient medium B5 containing 0.2 mg/L BAP, 0.5 mg/L IAA, and 322 mg/L putrescine [50].

IMB induction medium with 55 mg/L sucrose and 0.4 mg/L TDZ (developed by our group) made it possible to achieve (in culture) up to nine induction ovules (in Petri dish) of red beet in the Nezhnost’ genotype. Thidiazuron, as a derivative of phenylurea, belongs to the cytokinins group [68]. Moreover, phenylurea, similar to adenine cytokinins, showed the same biological activity: it increased callus growth, induced organogenesis, and stimulated ethylene formation. Shantz and Steward (1952) [69], working with carrot cells, found that coconut milk constituents stimulated cell division. They later determined that some of these constituents were phenylurea compounds. Phenylurea compounds, in particular thidiazuron, had greater activity than zeatin. According to the literature, the advantage of using thidiazuron is a significant stimulation of morphogenesis processes, including regeneration from meristematic tissues at low concentrations. Thidiazuron is an active growth regulator widely used in induction and regeneration nutrient media to enhance seedling responsiveness [70]. Initially, thidiazuron was used as a defoliant for cotton [71]. Thidiazuron has also been successfully used in place of cytokinins in tobacco cells and cotton callus cultures and for clonal micropropagation of woody species to enhance induction activity in cucumber ovule cultures [54,72,73].

Along with the action of cytokinins and auxins, the effect of light on plants in cell and tissue culture is a crucial morphogenic factor. Data from the literature report the cultivation of sugar beet ovules at different temperatures ranging from 24 to 30 °C [39,43,44] and red beet at 27 or 32 °C [48]. Furthermore, according to some reports, the incubation of isolated ovules can be performed at lower temperatures (25/18 °C during the day/night) in the dark [74]. Red beet ovules are cultured in light [50]. In most published studies on sugar beet gynogenesis, light treatment had little effect on the induction process [75]. Some authors studying sugar beet gynogenesis reported embryogenic induction in the dark [58]. Sufficiently successful results in our studies, in which induction of up to 25% of placed ovules was achieved, were obtained by culturing them in the dark at 28 °C.

Only 55% of red beet genotypes used in our studies were responsive at the induction stage in in vitro unpollinated ovule culture. This is in agreement with many published works indicating that genotype is one of the most important factors determining the yield of induced ovules in gynogenesis culture. Baranski (1996) [48] noted high genotypic dependence in the gynogenesis process of red beet; other authors researching sugar beet indicated differences between genotypes [29,31,39,57]. In a study by Lux et al. (1990) [38], the haploid yield was genotype-dependent and ranged from 0% to 13.7% with an average of 1.0%, and haploid plants could be obtained from approximately 50% of all studied genotypes.

When plants are cultivated in vitro in an enclosed space, ethylene accumulates in large quantities, which can have a strong morphogenetic effect on plant cells. Ethylene inhibits seedling elongation, prevents leaf growth and causes delayed mitosis, inhibits polar auxin transport, and promotes the formation of its conjugates. Apparently, this is the reason for ethylene’s ability to intensify the aging process; its production dramatically increases under stress and tissue damage. An increase in auxin and cytokinin concentrations also activates the production of ethylene. Ethylene’s mechanism of action has yet to be adequately studied. Presumably, it affects the cytoskeleton state, interconnection of membranes, microtubules, and microfilaments. All this leads to a decreased regenerative ability of explants [76]. Raldugina and Sobolkova (1994) [77] showed that ethylene accumulation in vessels inhibits the growth and development of rape plants. In *B. campestris*, high levels of ethylene caused vitrification, i.e., distortion in plant development, and also inhibited shoot formation [78,79]. On the other hand, ethylene does not always negatively affect cell growth and development processes. The presence of ethylene was found to stimulate elongation of axillary shoots in *Thuja occidentalis* L., as well as induce early embryoid development in *Picea glauca* [80].

Thus, ethylene plays an important role in the growth and differentiation of plant cells and tissues under in vitro conditions. Various substances, such as aminoethoxyvinylglycine, polyamines, nitrate, and silver sulfate inhibit ethylene synthesis in many crops, including various species that are difficult to propagate, e.g., rice, *B. campestris* [78,81,82], *B. juncea* [79,81], radish [80], maize [83], wheat [84], and consequently, stimulate shoot regeneration.

According to other literature data, silver ions in the form of nitrate are known to play an important role in influencing somatic embryogenesis, shoot formation, and effective root formation [85,86]. Silver ions are also used in the form of silver thiosulfate; thus, when stem segments of *B. juncea*, *B. campestris*, *B. napus*, *B. nigra,* and *B. carinata* were cultured, silver nitrate and silver thiosulfate increased regeneration activity [87]. Silver nitrate alters both the frequency of shoot formation and efficiency of ethylene production by binding ethylene receptors located primarily on the intracellular membrane and influencing cells’ ethylene-sensing mechanism [79,88,89].

In in vitro unpollinated ovule culture of sugar beet, Gurel et al. (2000) [43] described the inclusion of silver nitrate (at a concentration of 2.5 or 5 mg/L) on MS nutrient medium [65] either reduced the induction activity of sugar beet ovules (at a concentration of 2.5 mg/L) or completely inhibited it (at 5 mg/L). In this study, the authors hypothesized that ovules’ coloration was unchanged for a longer duration due to the fact that silver nitrate retarded ethylene-induced senescence. However, in contrast to these results, our study observed an increase in the yield of responsive red beet ovules from one culture when silver nitrate was added to all genotypes.

The effects of genotypic variability as well as the composition of regeneration nutrient medium impact not only the intensity of the induction process but also the frequency of the plant regeneration process from induced ovules. Thus, out of six beet genotypes responsive at the induction stage, three genotypes showed regenerative processes in the presence of 1 mg/L BAP and 0.1 mg/L GA_3_ leading to plant development. In the example of sugar beet, only from a small proportion of induced ovules was plant development possible with a frequency of 10.9% [30], 10% [57], 25% [38], 36.1% [90], 36.9% [63], and 43.8% [91].

In our studies, regenerant plants obtained from unpollinated ovule cultures of red beet were difficult to root. Apparently, prolonged cultivation of explants on regenerative nutrient media with cytokinins (BAP) resulted in their gradual accumulation in tissues above the required physiological level, which led not only to the formation of a large number of plants with altered morphology but also to a decrease in rooting ability. According to the literature, the effects of long-term exposure to growth regulators can negatively affect the rooting process [59,92]. Natural and synthetic auxins are widely used in plant cells, tissue, and organ cultures to induce specific morphogenetic responses [93]. The use of IBA for rooting, which allowed us to obtain in vivo acclimatized red beet plants, has been reported in a number of publications. Root formation, with a frequency from 0% to 65.2%, depending on the combination of growth regulators used for shoot regeneration, was observed in the cultivation of sugar beet shoots on MS medium with 14.8 µM IBA and 0.049 µM 2iP(6-(γ,γ-Dimethylallylamino)purine) [90]. The inclusion of IBA in nutrient medium in combination with 2ip was also reported by Goska (1997) [94].

Determination of the ploidy level of obtained plants in unpollinated ovule cultures is an important step in obtaining doubled haploids. Obtaining plants with a haploid set of chromosomes indicates a gynogenic origin of the derived plants. For sugar beet, the formation of haploids, in most cases, is known in unpollinated ovule cultures [38,46,63,94], although the formation of spontaneous doubled haploids and tetraploids of gynogenic origin has also been reported [58]. The spontaneous doubled haploids phenomenon is often found in all plant species and avoids the laborious diploidization step when obtaining doubled haploids, which requires treatment with antimitotic substances [95,96,97]. It is known that in sugar beet, only 2–10% of plants obtained in unpollinated ovule cultures spontaneously doubled their chromosome set [31,39,43,63,75,95]. In our study, all red beet plants obtained in an in vitro unpollinated ovule culture were haploid, which was confirmed by flow cytometry and direct chromosome counting. This confirmed that all obtained plants were of gynogenic origin.

## 4. Materials and Methods

### 4.1. Plant Material and Growing Conditions

Eleven cultivars from hybrid and varietal populations of red beet (*Beta vulgaris* L. ssp. *europaea* Krass. var. *atrorubra* Krass; synonyms: *Beta vulgaris* ssp. *rapaceae* var. *atrorubra* Krass; *Beta vulgaris* L. ssp. *europea* Krass. convar. *esculenta* Salisb. var. *rubra*) from the collection of the Laboratory of Breeding and Seed Production of Table Roots at the Federal State Budgetary Scientific Institution Federal Scientific Vegetable Center (FSBSI FSVC) were used in the present study.

Red beet plants were grown in winter glazed and spring polycarbonate greenhouses. Root crops, which underwent vernalization at 4 °C for 4–6 months, were planted in peat-soil mixture according to the scheme of planting 70 × 20 cm. Planting was carried out in two terms: in February and April. The donor plants were watered as needed and fertilized once a week with liquid commercial fertilizer Aquarin (Bui, Russia), containing N-13%, P_2_O_5_-5%, K_2_O-25%, MgO-2%, S-8%, Fe (EDTA). 0.054%, Zn (EDTA) −0.014%, Cu (EDTA) −0.01%, Mn (EDTA) −0.042%, Mo-0.004%, B-0.02%.

### 4.2. Sterilization of Explants

Buds were collected, and experiments were laid from the middle of May to August. Inflorescences 7–10 cm long were cut from donor plants and placed in a refrigerator in a humid chamber at 4–5 °C. Buds were selected from the spike-like peduncle, located on the peduncle section immediately after the newly opened flower (an inflorescence fragment with 8–10 buds 2 to 6 cm long, depending on the genotype).

Surface sterilization of collected buds was performed for 30 s in 96% ethanol solution, then for 15 min in 50% aqueous solution of the commercial preparation “Belizna” (contains 10% sodium hypochlorite) with the addition of Tween-20 (Panreac, Barcelona, Spain) (1 drop per 100 mL solution), then washed three times in sterile distilled water, in each batch for 10 min. The sterilized inflorescences were placed on damp filter paper and stored in sterile conditions in glass Petri dishes 11 cm in diameter until the isolation of the ovules.

### 4.3. Cultivation of Unpollinated Ovules In Vitro

After sterilization, ovules were isolated from buds using dissecting needles under a Stemi 305 stereomicroscope (Carl Zeiss Microscopy GmbH, Germany) at 10× magnification in a laminar box and placed in 94 × 16 mm sterile Petri dishes (Greiner Bio-One GmbH, Frickenhausen, Germany) containing 20 mL of induction culture medium.

For induction of gynogenesis, we used nutrient medium IMB (induction medium for *Beta vulgaris*—developed in the laboratory of biotechnology of FSBSI FSVC) containing 55 g/L sucrose (Table 4), 3 g/L phytogel, 200 mg/L ampicillin, and 0.4 mg/L TDZ.

In the experiment to study the effect of silver nitrate on the induction of gynogenesis, silver nitrate at a concentration of 22 mg/L was added to the nutrient medium IMB.

Petri dishes were placed in incubator Binder BF 260 BINDER GmbH (Tuttlingen, Germany) and cultured for four to six weeks in the dark at 28 °C for induction of gynogenesis until embryoids or callus emerged.

Cultivated ovules were observed and photodocumented every 3–7 days using a Stemi 508 stereomicroscope with an Axiocam 305 color camera (Carl Zeiss Microscopy GmbH, Germany).

### 4.4. Plant Regeneration

Embryoids and induced ovules with well-developed callus of different coloration (from white to intensely red) were transferred to solid MS nutrient medium with 1 mg/L BAP and 0.1 mg/L GA_3_. Subculture of callus structures was performed for 5–7 weeks until the formation of rosettes with leaves, transplantation to fresh nutrient media was performed every two to three weeks. Microrosettes with leaves were transferred to hormone-free MS medium with 2% sucrose, 3 g/L phytogel to form normally developed rosettes with well-developed 5–7 leaves. Cultivation was carried out on racks with mixed illumination with fluorescent lamps of two types: OSRAM Fluora L36W/77 (predominantly blue and red spectrum) and Philips 36W/54-765 (predominantly white spectrum), at a total illumination of 3000 lux, photoperiod of 16 h day and 8 h night at 25 °C around the clock.

### 4.5. Rooting of Regenerant Shoots

To induce in vitro rhizogenesis in red beet cv. Nezhnost’ microrosettes with well-developed 5–7 leaves were transferred to three different nutrient media:

(1) MS media without plant growth regulators supplemented with 2% sucrose, 3 g/L phytogel;
(2) ½ IMB supplemented with 2 mg/L IBA, 2% sucrose, 3 g/L phytogel;
(3) ½ IMB without plant growth regulators, supplemented with 2% sucrose and 3 g/L phytogel (basal part of microrosettes was placed in sterile concentrated IBA solution (50 mg/L) for 10–15 s before putting them on the nutrient medium). The second immersion in concentrated IBA solution was performed after two weeks. Transplantation to fresh nutrient medium was performed every two weeks. For rooting microrosettes obtained from cv. Dobrynya and b.a. 128, only a variant of nutrient medium № 3 was used.

### 4.6. Cultivation of Regenerating Plants

The rooted plants with normally developed leaves and root systems were transferred into growing vessels with a mixture of peat and perlite (7:3, vol./vol.) and covered with perforated plastic cups for acclimatization to in vivo conditions. The regenerated plants were grown in a vegetation chamber with a docking lamp (Plantastar HPS lamp, 600 W, Osram) at a constant temperature of 25 °C, illumination of 8000 lux, and a 16 h/8 h photoperiod. Weekly fertilization was carried out with a solution of commercial complex water-soluble fertilizer “Aquarin”.

### 4.7. Ploidy-Level Determination by Flow Cytometry

Materials for ploidy determination were prepared from young leaves of regenerated plants. Approximately 0.5–1.0 cm2 (20 mg) of young leaves was cut with a razor blade in 1 mL of lysis buffer (0.2 M Tris, 4 mM MgCl2, 50 µg/mL RNA-ase, 0.5% (*v/v*) TRITON X-100, 0.5% (*v*/*w*) polyvinylpyrrolidone K15, and 50 µg/mL propidium iodide, pH 7.5) [98]. Fluorescence data of isolated nuclei were detected using a Partec CyFlow PA flow cytometer (Partec, GmbH) with a 532 nm laser light source. Histograms were visualized and processed using the Flowing Software 2.5.1. software (University of Turku, Finland). Samples were filtered through a 50 µm nylon filter. The diploid (2n = 2x = 18) donor plants of red beet cv. Nezhnost’ were used as external standards to determine the ploidy. Visualization and plot constriction were performed using Flowing Software 2.5.1. (University of Turku, Turku, Finland). Data observed were calculated with XLStat software (https://www.xlstat.com/en/, accessed on 25 October 2021) (Addinsoft).

### 4.8. Ploidy-Level Determination by Chromosome Counting

Chromosome counting was performed on crushed preparations of stem meristem and root tips stained with the propion-lacmoid method. Fixation (in propionic acid) and staining (with lacmoide) of the material occurred simultaneously in a standard propionic-lacmoide solution for 24 h. To prepare propion-lacmoide, 5 g of lacmoide was added to 50 mL of 50% propionic acid and left in a dark place for 3–5 days, shaking the flask periodically but not heating it. After that, the solution was filtered into a dark glass vessel and stored in a dark place. Maceration of stained tissues was carried out by boiling in 40% propionic acid solution: stem meristem—20–30 s; large root meristem—45–60 s; small root meristem—20–30 s from the boiling point. After boiling, the material was allowed to cool for 1–3 min, and part of the material was transferred into a drop of 40% propionic acid on a slide, covered with a coverslip to prepare crushed preparations. For preservation, the preparation was fused with paraffin and left to differentiate for 30 min or more. The obtained images were processed and documented using Axio Vision software, version 4.8 (Carl Zeiss MicroImaging, Jena, Germany).

### 4.9. Statistical Analysis

Ovules induction capacity was determined as the average number of induced ovules per Petri dish. For each genotype and in the experiment with the addition of silver nitrate, isolated ovules were cultured in five Petri dishes. In each Petri dish, 35 ovules were cultured, which amounts to 175 ovules for each genotype. The yield of regenerated microshoots was determined as the average number of microshoots obtained from one ovule by five replications for each genotype.

Rooting efficiency was determined by the number of rooted shoots, expressed as a percentage, after 4 weeks of cultivation on rooting medium. The error of the sampling fraction of a percent was calculated by the formula Sp = √pq/N, where Sp—sampling fraction error; p, q—probability of occurrence of two characteristics in the total population (%); N—total number of plants [99].

Statistical analysis was performed using single-factor or two-factor analysis of variance (ANOVA), and mean values were compared using Student’s *t*-test with 95% probability. Statistical analysis was performed using Microsoft Excel 2010 for Windows 10.

## 5. Conclusions

This study showed the possibility of obtaining gynogenic red beet plants in an in vitro unpollinated ovule culture. An induction medium consisting of IMB with 0.4 mg/L thidiazuron was developed, allowing the number of responsive ovules per Petri dish to be as high as 25%. The stimulating induction of silver nitrate in one culture dish increased the number of responsive ovules by a maximum of 2.4 times. The regenerating nutrient medium (MS) with 20 g/L sucrose, 3 g/L phytogel, 1 mg/L BAP and 0.1 mg/L GA_3_ was found to be favorable for obtaining microshoots under in vitro conditions. The most efficient rooting method using IBA to produce plants acclimatized to ex vitro conditions was determined. The evaluation of gynogenic plants’ ploidy by flow cytometry and direct chromosome counting showed that all obtained gynogenic plants were haploid (2n = x = 9).

## Figures and Tables

**Table 1:** Induction and regeneration activity of different red beet genotypes in an unpollinated ovule culture in vitro on IMB medium with 0.4 mg/L TDZ.

Genotype	Average Number of Induced Ovules/Petri Dish	Maximum Number of Induced Ovules/Petri Dish	Average Number of Microshoots In Vitro/Ovule	Number of Plants Acclimatized to Ex Vitro Conditions
cv. Nezhnost’	7.4 ± 0.23 ^f^	9	4.9 ± 1.8 ^b^	10
cv. Dobrynya	5.2 ± 0.17 ^d^	6	3.3 ± 0.8 ^b^	3
b.a. P-155	1.6 ± 0.18 ^b,c^	3	0 ^a^	0
b.a. B-131	6.6 ± 0.18 ^e^	8	2.1 ± 0.5 ^b^	0
b.a. 135	1.2 ± 0.37 ^b^	2	0 ^a^	0
b.a. 128	2.0 ± 0.14 ^c^	3	2.3 ± 0.7 ^b^	2
b.a. 130	0 ^a^	0	0 ^a^	0
b.a. 132	0 ^a^	0	0 ^a^	0
b.a. 142	0 ^a^	0	0 ^a^	0
b.a. 148	0 ^a^	0	0 ^a^	0
b.a. 138	0 ^a^	0	0 ^a^	0

Note: For each genotype, average values with five repeats in each ± SE (standard error) are presented. Each Petri dish contains 35 ovules in in vitro culture. One-way analysis of variance (ANOVA) was used, and means were compared using Student’s *t*-test with 95% probability. Values marked with similar letters had no significant difference at *p* ≤ 0.05.

**Table 2:** Regeneration capacity of different red beet genotypes from induced ovules.

Genotype	Number of Induced Ovules	Type of Induction, Embryoid/Callus, Number of Ovules		Number Microrosetts Received	Number of Dead Microrosetts	Number of Microshoots Preserved in Culture In Vitro	Number of Microshoots with Roots
cv. Nezhnost’	37	Embryoid	9	19	11	5	3
		Callus	28	74	33	22	19
cv. Dobrynya	26	Embryoid	6	23	11	8	4
		Callus	20	48	23	13	12
b.a. B-131	33	Embryoid	5	9	9	0	0
		Callus	28	15	15	0	0
b.a. P-155	8	Embryoid	0	0	0	0	0
		Callus	8	0	0	0	0
b.a. 128	10	Embryoid	0	0	0	0	0
		Callus	10	28	14	8	6
b.a. 135	6	Embryoid	0	0	0	0	0
		Callus	6	0	0	0	0

**Table 3:** Rooting of red beet regenerant plants cv. Nezhnost’.

Variant of Nutrient Medium	Number of Microrosetts	Number of Rooted Microrosetts	Rooted Microrosetts, p ± Sp *, %
MS without plant growth regulators	20	1	5.0 ± 4.9
½ IMB + 2 mg/L IBA	20	5	25.0 ± 9.7
½ IMB without plant growth regulators + IBA (immersion)	20	16	80.0 ± 8.9

Note: * p—the share of rooted plants (%), Sp—sampling fraction error (%).

**Table 4:** Composition of nutrient mediums used for obtained red beet gynogenic plants in culture of unpollinated ovules in vitro.

Nutrient Medium Components	Concentration of Components in the Nutrient Medium (mg/L)		
	Induction Medium IMB (Induction Medium For *Beta vulgaris*)	Regeneration Medium MS (Murashige, Skoog, 1962)	Rooting Medium 1/2 IMB
NH_4_NO_3_	825.000	1650.0	412.500
(NH_4_)_2_SO_4_	67.000	-	33.500
NaH_2_PO_4_ ·2H_2_O	84.800	-	42.400
KNO_3_	2200.000	190.000	1100.000
CaCl_2_	222.700	332.200	111.350
MgSO4·7H_2_O	310.000	371.000	155.000
KH_2_PO_4_	85.000	170.000	42.500
Na_2_EDTA·2H_2_O	37.300	37.300	18.650
FeSO_4_ 7H_2_O	27.800	27.800	13.900
H_3_BO_3_	4.600	6.200	2.300
MnSO_4_·5H_2_O	19.200	24.100	9.600
ZnSO_4_·7H_2_O	6.300	10.600	3.150
KI	0.790	0.830	0.395
Na_2_MoO_4_·2H_2_O	0.250	0.250	0.125
CuSO4·5H2O	0.025	0.025	0.012
CoCl_2_·6H_2_O	0.025	0.025	0.012
Thiamine-HCl (B1)	5.050	0.100	2.525
Pyridoxine-HCl (B6)	0.750	0.500	0.375
Nicotinic acid	0.750	0.500	0.375
Glycine	1.000	2.000	0.500
Myo-Inositol	100.000	100.000	50.000
L-Glutamine	800.000	-	-
Glutathione	30.000	-	-
L-Serine	100.000	-	-
Sucrose	55,000.000	20,000.000	20,000.000

## Data Availability

Not applicable.
